# Gastric Necrosis due to Acute Massive Gastric Dilatation

**DOI:** 10.1155/2013/847238

**Published:** 2013-07-28

**Authors:** Ibrahim Aydin, Ahmet Pergel, Ahmet Fikret Yucel, Dursun Ali Sahin, Ender Ozer

**Affiliations:** Department of Surgery, School of Medicine, Recep Tayyip Erdogan University, 53100 Rize, Turkey

## Abstract

Gastric necrosis due to acute massive gastric dilatation is relatively rare. Vascular reasons, herniation, volvulus, acute gastric dilatation, anorexia, and bulimia nervosa play a role in the etiology of the disease. Early diagnosis and treatment are highly important as the associated morbidity and mortality rates are high. In this case report, we present a case of gastric necrosis due to acute gastric dilatation accompanied with the relevant literature.

## 1. Introduction

The pathogenesis of acute gastric dilatation (AGD) is not fully known, and several theories currently exist. Vascular compression, herniation, volvulus, acute necrotizing gastritis, complications after abdominal surgery, anorexia, bulimia nervosa, trauma, exposure to caustic materials, diabetes, and acute massive gastric dilatation are indicated in the etiology of the disease [1]. 

Gastric ischemia and necrosis are rare due to the rich collateral blood flow to the stomach. However, if a sudden increase in gastric pressure occurs, gastric ischemia and necrosis can develop due to the impairment of the intramural blood flow [2].

In this case report, we present a case of gastric necrosis due to acute gastric dilatation in terms of the literature.

## 2. Case Report

**Figure 1:**
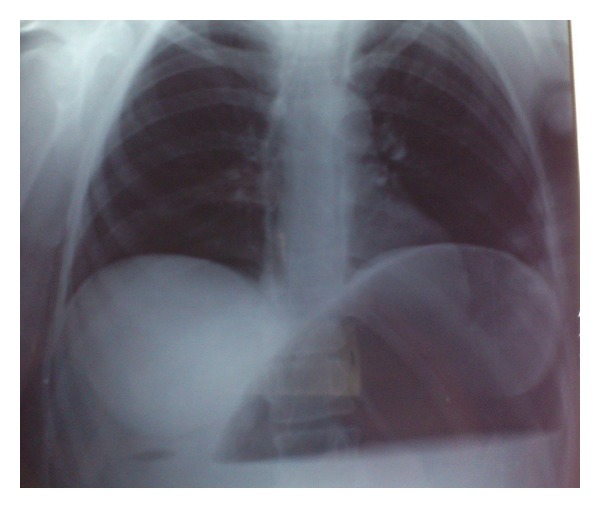
Direct abdominal X-ray image, obtained in a standing position, showing air almost completely filling the left upper quadrant of the fundus and extending into the right upper quadrant.

**Figure 2:**
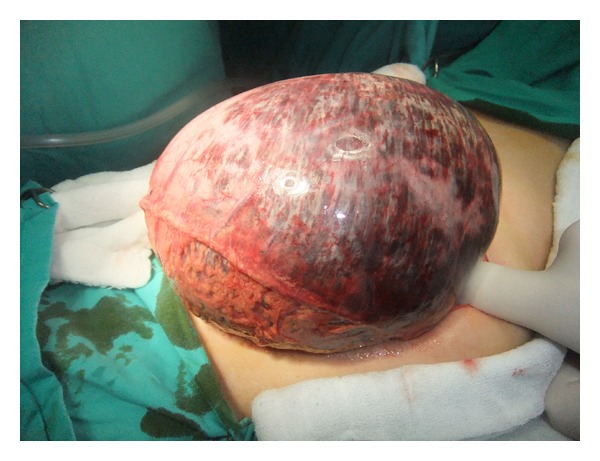
Diffuse gastric dilatation.

**Figure 3:**
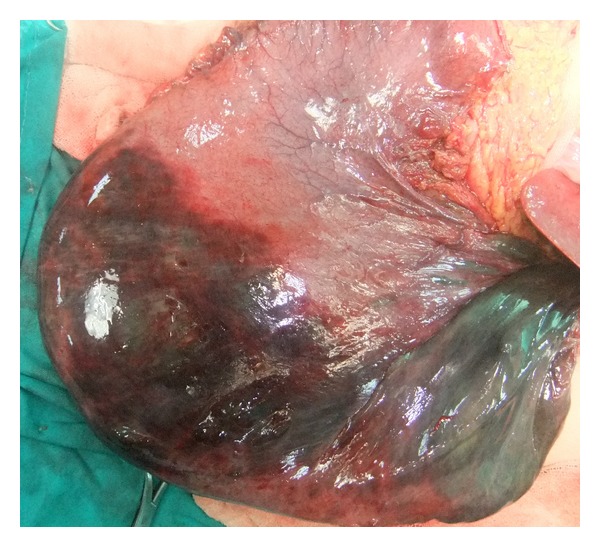
Diffuse necrotic zones in the gastric fundus and greater curvature of the corpus.

A 26-year-old hemiplegic, mentally and motor-retarded female patient was admitted to the ER with sudden-onset abdominal pain, abdominal swelling, and vomiting. The patient had a temperature of 38.5°C, blood pressure of 80/60 mmHg, heart rate of 112/min, and respiratory rate of 32/min. Physical examination revealed abdominal distention, defense, rebound, and tenderness. Her laboratory tests were normal except for elevated white blood cell count (25.000/mm³). Direct abdominal X-ray images obtained in a standing position showed air almost completely filling the left upper quadrant of the fundus and extending into the right upper quadrant (Figure 1). Immediately after the NG tube was inserted, 2000 cc of gastric dilatation fluid (coffeeground colored fluid) was discharged. The patient was prepared for urgent surgery. The patient's stomach was highly dilated during exploration (Figure 2). Diffuse necrotic zones in the gastric fundus and greater curvature of the corpus were detected (Figure 3). About 3000 cc of fluid was aspirated from the stomach during surgery. Total gastrectomy and esophagojejunostomy were performed on the patient, and she was discharged ten days after the operation.

## 3. Discussion

Gastric necrosis due to rich blood flow to the stomach is relatively rare. Acute gastric dilatation was first described by Todd et al. in 2000 [3]. Casper et al. identified AGD in only four cases in their study of 23,000 postmortem cases [4]. The causes of gastric infarct include intrathoracic herniation, volvulus, acute necrotizing gastritis, drinking corrosive substances, vascular compression, and AGD [1, 5]. AGD has also been found to occur in patients with anorexia nervosa, bulimia, low body mass index, or nutritional deficiency [6]. Additionally, in 1859, Brinton suggested the atonic theory: that gastric atonia and muscular atrophy occur in patients with eating disorders during hunger; then, sudden and excessive eating causes the weakened stomach to overload [7]. Since no undigested food was present in the stomach, only excessive gastric dilatation, we hypothesized that gastric necrosis played a role in the etiology of AGD in the present case.

Evaluation of psychiatric patients with abdominal pain is difficult. A detailed anamnesis cannot be obtained, and thus, the surgeon must act based on physical examination findings and must be informed of and experienced with surgical diseases seen in psychiatric patients. Physical examination findings can widely differ amongst patients. Diagnosis can be supported by radiological imaging of gastric dilatation and pneumoperitoneum [8]. Resuscitation and surgical treatment are necessary in this life-threatening condition. The mortality rate can be as high as 80% in cases of incorrect diagnosis and delayed treatment [9]. Our case was a hemiplegic, mentally and motor-retarded patient, and the diagnosis was based on physical examination and direct abdominal X-ray findings.

 Acute gastric dilatation occurs mostly in women (67%) and is frequently found in the lesser curvature of the stomach [10]. AGD is also associated with a considerably high mortality rate (73%) [5]. In the present case, gastric necrosis due to AGD was detected in the greater curvature, fundus, and corpus of the stomach.

Resection of the gangrenous gastric part is essential in AGD. Usually, a total gastrectomy is required. Resection and esophagojejunostomy are successful under optimal conditions [3, 9]. In cases of delayed treatment or with generalized peritonitis, decompression and drainage are recommended in addition to resection [11]. Total gastrectomy and esophagojejunostomy were performed on our case because necrosis was present in the fundus and greater curvature of the corpus and perforation was absent. 

In conclusion, early diagnosis of gastric necrosis due to AGD is highly important due to the associated high morbidity and mortality rates. Gastric necrosis due to AGD must be considered in differential diagnosis to avoid late diagnosis in mentally retarded patients and patients with eating disorders.
